# Ventral Tegmental Area Afferents and Drug-Dependent Behaviors

**DOI:** 10.3389/fpsyt.2016.00030

**Published:** 2016-03-07

**Authors:** Idaira Oliva, Matthew J. Wanat

**Affiliations:** ^1^Department of Biology, Neurosciences Institute, University of Texas at San Antonio, San Antonio, TX, USA

**Keywords:** VTA, substance use disorders, addiction, dopamine, plasticity

## Abstract

Drug-related behaviors in both humans and rodents are commonly thought to arise from aberrant learning processes. Preclinical studies demonstrate that the acquisition and expression of many drug-dependent behaviors involves the ventral tegmental area (VTA), a midbrain structure comprised of dopamine, GABA, and glutamate neurons. Drug experience alters the excitatory and inhibitory synaptic input onto VTA dopamine neurons, suggesting a critical role for VTA afferents in mediating the effects of drugs. In this review, we present evidence implicating the VTA in drug-related behaviors, highlight the diversity of neuronal populations in the VTA, and discuss the behavioral effects of selectively manipulating VTA afferents. Future experiments are needed to determine which VTA afferents and what neuronal populations in the VTA mediate specific drug-dependent behaviors. Further studies are also necessary for identifying the afferent-specific synaptic alterations onto dopamine and non-dopamine neurons in the VTA following drug administration. The identification of neural circuits and adaptations involved with drug-dependent behaviors can highlight potential neural targets for pharmacological and deep brain stimulation interventions to treat substance abuse disorders.

## Introduction

Illicit drug use is a significant global problem, with the United Nations Office on Drugs and Crime estimating that 246 million people worldwide used illicit drugs in 2013. More problematic is the high incidence of substance use disorders (SUDs), which in 2014 was estimated to afflict roughly 21.5 million people in the US, corresponding to ~8% of the population (1). In addition to the personal impact of a SUD, there is a significant economic impact due to lost productivity, crime, and health care costs, which according to the US office of National Drug Policy is estimated to cost $180.8 billion per year in the US alone.

SUDs are now recognized to exist along a continuum where the severity of the disorder is related to the number of diagnostic criteria met by an individual within the past year. According to the DSM-V, the criteria for a SUD fall into four major symptomatic clusters: impaired control (i.e., use more than intended), social impairment (i.e., substance use at the expense of personal relationships and impaired job performance), risky behavior (i.e., use despite known adverse consequences), and pharmacological effects (i.e., tolerance and withdrawal). One of the most daunting aspects in treating SUDs is the high incidence of relapse, which occurs in ~40–60% of individuals (2). In drug users, exposure to drug-paired cues elicits craving that in turn can promote the possibility of a relapsing episode (3). Weakening the relationship between drugs and associated cues holds promise as a non-pharmacological method for treating SUDs (4). However, our understanding of the specific neural circuits and neural adaptations responsible for drug-related behaviors is incomplete.

## Rodent Models of Drug-Dependent Behaviors

Rodent model systems are commonly employed to examine the effects of abused drugs on behavior. In this review, we will concentrate on psychostimulants and opiates, as extensive laboratory research has focused on these drug categories. The non-contingent administration of psychostimulants or opiates increases locomotor activity in rodents (5). Repeated non-contingent drug injections can lead to a progressive and long-lasting increase in this drug-induced locomotor activity, a phenomenon referred to as behavioral sensitization (5). A single injection of cocaine at high doses is also capable of eliciting sensitization (6, 7). Furthermore, even when no drug is administered, locomotor activity is elevated in the same context where animals received a single drug injection on the preceding day (8). These results illustrate that the association between a drug and the context where the drug is experienced is rapidly learned following a single exposure.

Drug-paired cues exert a powerful influence over behavioral actions in individuals with a SUD (3). The development of an association between drugs and cues can be examined in humans in the laboratory (9, 10), as well as in rodents by utilizing a conditioned place preference (CPP) behavioral paradigm (11). This rodent assay involves repeated non-contingent drug injections in one chamber and control injections in an adjacent, but contextually distinct chamber. The relative preference between the drug-paired and control contexts is subsequently assessed in a test session where the rodent can freely access both chambers in a drug-free state (11). The CPP training procedure can include an extinction phase and a reactivation test (12, 13), which models drug abstention and relapse observed in humans suffering from a SUD. While CPP paradigms examine contextual learning involving reinforcing outcomes, conditioned place aversion (CPA) assays examine learning involving aversive outcomes. In particular, CPA paradigms are commonly utilized to study the negative affective state following drug withdrawal (14, 15).

Behavioral sensitization and CPP paradigms are relatively easy to implement, but they require experimenter administered drug injections. Rodents can be readily trained to self-administer drugs via an intravenous catheter. A number of drug self-administration assays have been developed to model the behavioral symptoms observed in humans with a SUD. For example, rodents with limited access (1 h) to drugs in daily self-administration sessions maintain stable drug intake. However, rodents with extended access (6 h) to drugs increase their intake over multiple training sessions, similar to the escalated drug consumption that can be observed in individuals diagnosed with a SUD (16–18). Just as drug use does not necessarily lead to a SUD, not every rodent who self-administers drugs will develop an addiction-related phenotype. When rodents are extensively trained to self-administer drugs (~3 months), a subset of rats exhibit characteristics found in humans with SUDs, such as persistent drug seeking in the absence of reinforcement, exerting greater effort to obtain a drug infusion, and seeking drugs despite aversive consequences (19). Rodents trained to self-administer drugs are also used to model relapse. Relapse in humans is often precipitated by three major factors: taking the drug, exposure to cues previously associated with the drug, or experiencing a stressful life-event (20–22). These same triggers (drug intake, exposure to drug-related cues, or stress) can reinstate drug-seeking behaviors in rodent drug self-administration models as well (23).

Just as with humans with a SUD, drug-dependent behaviors in rodents involve a component of learning, whether it is contextual (behavioral sensitization, CPP, CPA, and cue-induced reinstatement) or operant (drug self-administration). While numerous brain regions are involved with mediating learning and drug-related behaviors, we will focus on the ventral tegmental area (VTA) in this review. We will also discuss the major inputs to the VTA, how these inputs influence VTA neuron activity, and present recent findings on how these VTA afferents are involved with drug-dependent behaviors.

## VTA Involvement in Drug-Dependent Behaviors

The dopamine neurons arising from the VTA that project to the nucleus accumbens (NAc) are involved with mediating the reinforcing actions of abused substances (24–26). While abused drugs increase dopamine levels in the NAc (27, 28), many non-habit forming drugs do not affect dopamine overflow (27). Psychostimulants affect dopamine levels primarily by altering dopamine clearance from the extracellular space (29, 30), whereas opiates indirectly elevate dopamine transmission by suppressing inhibitory input onto dopamine neurons (31–33).

The neural circuitry mediating any behavior is complex, though extensive research over the past few decades illustrates that the VTA is critically involved with both rewarding and aversive drug-dependent behaviors. For example, the VTA is required for behavioral sensitization induced by amphetamine or mu-opioid receptor agonists, though evidence for the involvement of the VTA in cocaine behavioral sensitization is mixed (5). The VTA is also involved with CPP for both psychostimulants and opiates (34–39), and with CPA elicited by kappa opioid receptor activation (15). The VTA is also necessary for stress-, cue-, and drug-primed reinstatement in rodents self-administering cocaine (23, 40–42) or heroin (43–45). While VTA-dependent behaviors are often mediated by dopamine neurons, increasing evidence illustrates the involvement of non-dopamine VTA neurons in regulating behavioral outcomes.

## Diverse Neuronal Populations within the VTA

The VTA along with the neighboring substantia nigra pars compacta are the primary dopamine producing nuclei in the brain (46). Early electrophysiological recordings indicated that the VTA was comprised of two distinct neuronal populations, presumed to be dopamine neurons and local GABA interneurons (31, 47). However, a subset of VTA neurons exhibited a unique electrophysiological response to serotonin and opioid receptor agonists, providing evidence for the existence of an additional neuronal population in the VTA (48). Accumulating evidence over the past decade has highlighted the complexity of the VTA both in regards to neuronal composition and projection targets.

Dopamine neurons comprise the largest neuronal population within the VTA, as tyrosine hydroxylase (TH), the rate-limiting enzyme for dopamine synthesis, is found in ~60% of VTA neurons (46, 49). VTA dopamine neurons typically innervate only a single target region, with different populations projecting to numerous brain nuclei, including the NAc, dorsal striatum, cortex, amygdala, globus pallidus, and lateral habenula (LHb) (46, 50, 51). However, recent evidence indicates that dopamine neurons projecting to the medial NAc also send collaterals outside of the striatum (50). Traditionally, dopamine neurons have also been identified based upon electrophysiological properties, including the presence of a long triphasic action potential, a low baseline firing rate, burst firing, and the presence of the *I*_h_ current (52, 53). However, action potential duration may not be sufficient to identify the neurotransmitter content of VTA neurons (49, 54). Additionally, many neurons within the medial aspects of the VTA have *I*_h_ but do not contain TH. While action potential duration and *I*_h_ are not always indicative of dopamine content, these electrophysiological properties can be related to where VTA neurons project (55–57).

The second largest neuronal population in the VTA consists of GABA neurons (~25%) that are commonly identified by the presence of glutamic acid decarboxylase (GAD) (58, 59). While initially thought to function primarily as local interneurons (31), VTA GABA neurons directly influence the activity of VTA dopamine neurons (60, 61) and also project to the ventral pallidum (VP), lateral hypothalamus (LH), and LHb, with smaller projections to the amygdala, prefrontal cortex (PFC), and NAc (62–64). Recently, dopamine neurons were identified as an additional source of GABA in the VTA, as these neurons can synthesize GABA through an aldehyde dehydrogenase-mediated pathway (65). VTA and substantia nigra dopamine neurons package GABA into vesicles through the vesicular transporter for dopamine, indicating that GABA can be coreleased with dopamine to elicit electrophysiological effects on medium spiny neurons in both the NAc and dorsal striatum (66, 67).

In addition to dopamine and GABA neurons, a small percentage of VTA neurons contain vesicular glutamate transporter 2 (VGluT2), a marker for glutamate neurons. These neurons predominately reside in the medial aspects of the VTA and project to the ventral striatum, PFC, VP, amygdala, and LHb, as well as synapse onto local dopamine neurons (57, 64, 68–72). A subset of the VGluT2 positive neurons in the VTA also express TH and can project to the PFC and ventral striatum (70). These neurons release both dopamine and glutamate (73–77) though they are not typically released at the same site or from the same synaptic vesicles (78). While the VTA was thought to be comprised solely of dopamine and GABA neurons, recent studies illustrate that the VTA is comprised of dopamine neurons that can corelease GABA, dopamine neurons that corelease glutamate, GABA neurons, and glutamate neurons.

Optogenetic modulation of VTA neurons can elicit either appetitive or aversive behavioral outcomes depending upon the neuronal population that is targeted. Activation of dopamine neurons is acutely reinforcing and sufficient for establishing a CPP, whereas silencing dopamine neurons is aversive and elicits a CPA (60, 79, 80). Stimulating VTA dopamine neurons also enhances reinforcing behaviors in operant tasks (81–84). In contrast, selective activation of VTA GABA neurons is aversive, elicits a CPA, and reduces reward consumption by inhibiting the activity of local VTA dopamine neurons (60, 61). Interestingly, activating VTA GABA neurons that synapse onto cholinergic interneurons in the NAc enhances the discrimination between neutral and aversive stimuli (63). Optogenetic activation of VGluT2-containing neurons in the VTA is also sufficient for establishing CPP, an effect that is mediated by activating local VTA dopamine neurons (72). Collectively, these studies suggest that VTA-mediated behavioral effects, including drug-dependent behaviors, likely involve a complex interplay between the distinct neuronal populations in the VTA.

## Afferent Regulation of the VTA

The VTA is innervated by a diverse array of inputs, many of which are interconnected. Large afferents to the VTA include the rostromedial tegmental nucleus (RMTg), VP, bed nucleus of the stria terminalis (BNST), LH, pedunculopontine tegmental nucleus (PPT), laterodorsal tegmental nucleus (LDT), dorsal raphe nucleus (DR), NAc, PFC, and amygdala (50, 85–87). While VTA dopamine and GABA neurons are innervated by many of the same brain regions (50), little is known about the inputs to VGluT2 positive neurons in the VTA. Below, we will discuss how notable inputs to the VTA can influence the activity of VTA neurons, how these inputs influence VTA-dependent behaviors, and recent findings on VTA afferents involved with drug-dependent behaviors.

### Rostromedial Tegmental Nucleus

The RMTg (also referred to as the tail of the VTA) is a nucleus comprised of GABA neurons that function as an inhibitory relay between the LHb and the VTA (86, 88–92). Lesions of the RMTg demonstrate a critical role for this brain region in modulating aversive behaviors (86). Additionally, neurons in the RMTg are activated by aversive stimuli and inhibited by rewards (86). The RMTg heavily influences the firing of VTA neurons, as RMTg inactivation increases dopamine neuron firing (93), whereas stimulating the RMTg attenuates dopamine neuron firing (93–95).

The RMTg is increasingly recognized as an important nucleus in mediating the effects of abused drugs. The reinforcing effect of opiates was originally thought to arise from activation of mu-opioid receptors on VTA GABA interneurons (31), though accumulating evidence suggests the major target of opiates is instead the RMTg afferents to the VTA (33, 96, 97). The administration of morphine decreases RMTg cell firing, which reduces the inhibition onto VTA dopamine neurons, resulting in elevated dopamine neuron firing (94–96). Indeed, selective activation of mu-opioid receptors in RMTg neurons projecting to the VTA is sufficient for eliciting a real-time place preference (98). Following opiate withdrawal, inhibiting RMTg neurons no longer elevates VTA dopamine neuron firing. This inability of the RMTg to disinhibit dopamine neurons is mediated in part by an alteration in VTA glutamatergic tone (93). While the RMTg projection to the VTA mediates the acute reinforcing effects of opiates (33, 96, 98), additional VTA afferent pathways are involved with dopamine neuron tolerance to opiates following withdrawal (93).

Psychostimulants also influence the activity of RMTg neurons (94). The non-contingent administration of cocaine elevates the levels of Fos, a transcription factor associated with increased neuronal activity, in RMTg neurons (99, 100). Interestingly, Fos levels in RMTg neurons projecting to the VTA are elevated following extinction in rats self-administering cocaine (101). The RMTg is also necessary for cocaine-related aversive behaviors that are observed once the rewarding effect of cocaine dissipates (102). Further experimentation is needed to validate whether the RMTg projection to the VTA is involved with both aversive and reinforcing behaviors elicited by cocaine.

### Ventral Pallidum

The VP is involved in processing rewarding stimuli and motivated behavior (103). GABA neurons in the VP provide a large source of inhibitory input to the VTA (87, 104). Activating VP neuron terminals elicits inhibitory GABA currents in both dopamine and non-dopamine VTA neurons (105). The functional effect of inactivating the VP results in an increase in the population activity in putative dopamine neurons (106) though the effect on non-dopamine VTA neurons is unknown. Numerous lines of evidence implicate the VP in drug-dependent behaviors. VP neurons projecting onto dopamine and non-dopamine neurons are acutely inhibited by opiates (105). Additionally, VP lesions or pharmacological manipulations in the VP can block morphine-induced sensitization (107, 108), drug-induced CPP (35, 109, 110), self-administration (111), and reinstatement (40, 41, 112). VP neurons projecting to the VTA are Fos activated following cue-induced reinstatement for cocaine (101) and silencing these neurons is sufficient for blocking cue-induced reinstatement (113). While VP neurons project to both dopamine and non-dopamine neurons in the VTA (105), it is unclear what neuronal population(s) in the VTA are influenced by the VP inputs during drug-dependent behaviors.

### Bed Nucleus of the Stria Terminalis

The BNST is involved in mediating fear and anxiety (114–120) and is considered to be a relay nucleus between stress and reward pathways (121, 122). The neuronal composition of the BNST is diverse, with efferent populations of GABA and glutamate neurons along with local GABA and cholinergic interneurons (122, 123). BNST neurons also express an assortment of neuropeptides including neuropeptide Y, corticotropin-releasing factor, enkephalin, dynorphin, and substance P (124). Electrical stimulation of the BNST exerts an excitatory influence on midbrain dopamine neurons (122, 125, 126) and elevates dopamine release in the NAc (127). Recent studies suggest that this excitatory effect on dopamine neurons is predominately mediated through GABA BNST neurons disinhibiting VTA GABA neurons, resulting in anxiolytic and rewarding behavioral outcomes (128–130). Interesting, glutamate neurons in the BNST also innervate VTA GABA neurons, and activation of these neurons elicits aversive and anxiogenic behaviors (129). Within the context of drug-dependent behaviors, local pharmacological manipulations illustrate a critical role of the BNST in the stress-induced reinstatement of drug seeking (41, 131, 132). Furthermore, recent studies implicate the BNST–VTA pathway in the locomotor-activating effects of cocaine (133) and in the expression of cocaine CPP (134), though the involvement of this pathway in other drug-dependent behaviors has not yet been explored.

### Lateral Hypothalamus

The LH is critical for the expression of motivated behaviors including feeding and drug seeking (135). The LH provides both glutamate and GABA inputs to the VTA (85, 136). In addition, LH neurons projecting to the VTA also contain neuropeptides such as neurotensin and orexin/hypocretin (137, 138). Electrical stimulation of the LH increases the activity of putative dopamine neurons and inhibits the activity of putative GABA neurons in the VTA (139). Many lines of evidence demonstrate that activation of this LH–VTA pathway is reinforcing. Rodents will readily self-stimulate for electrical activation of the LH, but this behavioral effect is inhibited by dopamine receptor antagonism (140) or inactivation of the VTA (141). Furthermore, optogenetic activation of LH inputs to the VTA also supports self-stimulation through a neurotensin-dependent mechanism (142).

Accumulating evidence over the past decade highlights the importance of orexin-containing neurons in feeding, the sleep/wake cycle, and drug-dependent behaviors (143). Orexin-producing neurons are exclusively localized within the hypothalamus and project widely throughout the brain (144), though it is the projection to the VTA that is heavily involved with drug-dependent behaviors. Intra-VTA injections of orexin receptor antagonists attenuate morphine CPP (145, 146), which is consistent with the reduced morphine dependence observed in orexin-deficient mice (147). Conversely, intra-VTA administration of orexin reinstates morphine CPP (12). Orexin antagonists targeting the VTA also diminish behavioral sensitization to cocaine (148), cocaine self-administration (149), and cue-induced reinstatement (150). Interestingly, orexin neurons in the LH also contain dynorphin, which inhibits the activity of VTA dopamine neurons. A recent study suggests that orexin in the VTA facilitates drug-related behaviors in part through attenuating the effects of dynorphin (149). Although the orexin-containing neurons in the LH have received considerable attention in the context of addiction, additional neuronal populations in the LH–VTA pathway are also likely involved in drug-dependent behaviors, as the non-orexin-producing neurons in the LH are Fos activated following cue-induced reinstatement (101).

### Laterodorsal Tegmental Nucleus and Pedunculopontine Tegmental Nucleus

The LDT and PPT are involved in modulating arousal and reward-driven behaviors (92, 151–154). These nuclei are comprised of distinct populations of acetylcholine, GABA, and glutamate neurons that project to the midbrain dopamine system (155, 156). Anatomical studies indicate that the VTA primarily receives input from the LDT (87, 155, 157). *In vivo* electrophysiological experiments illustrate that electrical stimulation of the LDT elicits burst firing in putative VTA dopamine neurons (158). Selective activation of LDT inputs to the VTA evokes excitatory currents in VTA dopamine neurons projecting to the lateral NAc (92). Stimulating this LDT–VTA pathway *in vivo* elicits CPP and reinforces operant responding (92, 154). Increasing evidence indicates that the LDT is also involved in drug-dependent behaviors. Specifically, local pharmacological manipulations demonstrate the LDT is critical for the acquisition and expression of cocaine CPP (159), as well as with cocaine-primed reinstatement of drug seeking (160). Interestingly, the cholinergic neurons of the LDT are involved with the behavioral responsiveness to cocaine-paired cues (161). Further studies are needed to ascertain whether drug-dependent behaviors also involve the GABA and glutamate projections from the LDT to the VTA.

Whereas the VTA is preferentially innervated by the LDT, the PPT primarily targets the substantia nigra (87, 155). Although the anatomical evidence indicates there is a small PPT projection to the VTA (87, 155), electrophysiological studies *in vivo* and *in vitro* suggest a functional relationship exists between the PPT and VTA (106, 162, 163). The discrepancy between the anatomical and electrophysiological studies is unclear, though proposed explanations include the possibility that a single PPT neuron innervates numerous VTA neurons or that electrical stimulation excites fibers of passage or nearby regions, such as the LDT (87). Regardless, electrical stimulations targeting the PPT increases burst firing of putative VTA dopamine neurons (106), while PPT inactivation reduces dopamine neuron firing to salient stimuli (162). The PPT is also implicated in drug-dependent behaviors, as lesions attenuate amphetamine- and morphine-induced locomotor activity (164), and PPT inactivation reduces cocaine-primed reinstatement of drug seeking (160). PPT lesions reduce both heroin self-administration and morphine CPP (165, 166). However, PPT cholinergic neurons are not involved with cocaine self-administration, heroin self-administration, cocaine CPP, and heroin CPP (167), suggesting the involvement of PPT glutamate and/or GABA neurons in these drug-related behaviors.

### Dorsal Raphe

The DR is the primary source of serotonin in the brain, but also contains glutamate (85), GABA (168), and dopamine neurons (169). While the DR is often studied within the context of controlling affective state (170), it is also involved in reinforcing instrumental behavior (171). Serotonin exerts a variety of electrophysiological responses in VTA neurons. The predominant *in vitro* response in putative dopamine neurons is excitatory, though a small proportion of dopamine neurons are inhibited by serotonin (172). In contrast, equal numbers of putative GABA neurons are excited and inhibited by serotonin (172). The net effect of these electrophysiological responses appears to be excitatory, as *in vivo* intra-VTA administration of serotonin elevates dopamine levels in the NAc (173).

Serotonin influences drug-related behaviors (174), which could involve the DR serotonin neurons projecting to the VTA. However, the DR projection to the VTA is primarily comprised of glutamate neurons that predominantly innervate dopamine neurons (85, 87, 175). Activation of DR glutamate neurons evokes excitatory currents in VTA dopamine neurons and elicits dopamine release in the NAc (175). Selective activation of the non-serotonergic DR–VTA pathway reinforces instrumental behavior and is sufficient for eliciting CPP (175, 176). In contrast, activation of serotonergic DR neurons projecting to the VTA is only weakly reinforcing (176). These anatomical and behavioral findings suggest that the VTA is likely not a primary locus where serotonin acts to influence drug-related behaviors. Instead, the non-serotonergic DR neurons projecting to the VTA are well positioned to mediate drug-dependent behaviors, though this has not yet been experimentally examined.

### Nucleus Accumbens

GABA neurons in the NAc project to the VTA and are thought to mediate a “long-loop” inhibitory feedback to regulate dopamine neuron activity (177). Mu-opioid receptor agonists acutely inhibit the GABA afferents from the NAc to the VTA (33, 178). The inhibitory transmission from the NAc inputs onto VTA GABA neurons is enhanced following repeated injections of cocaine, which in turn disinhibits VTA dopamine neurons (179). In addition to being influenced by opiates and psychostimulants, the NAc afferents to the VTA are Fos activated during cocaine cue-induced reinstatement (101). While these results suggest the NAc–VTA pathway is involved in drug-related behaviors, no experiments to date have examined the behavioral effect of selectively perturbing this pathway.

### Prefrontal Cortex

The medial PFC mediates a variety of cognitive functions (180), is involved in the reinstatement of drug-seeking behavior (23), and exhibits Fos activation following an acute administration of amphetamine (181). The VTA receives a dense glutamate projection from the medial PFC (85), with pyramidal neurons synapsing onto both dopamine and non-dopamine VTA neurons (62, 182). Electrically stimulating the PFC can either inhibit or excite putative dopamine neurons within the VTA (183, 184). Whereas single pulse or low frequency PFC stimulation inhibits a majority of VTA dopamine neurons (183–185), burst stimulation of the PFC excites >90% of VTA dopamine neurons (184). The mechanism behind the dopamine neuron excitation is unclear, as VTA dopamine neurons receive sparse input from the PFC (87, 186), with <15% of VTA dopamine neurons being excited by selective activation of medial PFC inputs (50). These findings collectively suggest the medial PFC preferentially targets VTA GABA neurons, though the relevance of this PFC-VTA pathway in drug-dependent behaviors has not been examined.

### Amygdala

The amygdala is an interconnected group of nuclei involved with attributing emotional value to cues (187, 188). The VTA receives amygdala input arising from the central nucleus of the amygdala (CeA) subdivision (87, 189). The CeA contains predominantly GABA neurons and is involved with fear conditioning (187, 188, 190), as well as with mediating the general motivational influence of rewarding cues (191, 192). In the context of drug-dependent behaviors, the CeA facilitates the expression of conditioned responding (193) and is also involved with mediating stress-induced reinstatement of drug-seeking behavior (194, 195). While the CeA projects to the VTA, it is currently unknown how this pathway influences VTA neuron activity and whether it is crucial for drug-dependent behaviors.

## Drug-Induced Synaptic Plasticity on VTA Neurons

The transition of an individual from drug naive or casual drug user to SUDs involves changes in the function of specific neural circuits (196). Given the importance of the VTA in drug-related behaviors, the synaptic adaptations in VTA dopamine neurons have been extensively studied and reviewed elsewhere (197–201). Numerous studies from a variety of laboratories have consistently demonstrated an increase in excitatory synaptic strength onto VTA dopamine neurons after *in vivo* exposure to abused drugs (202–208). Many of these studies examined the effect of drugs on the ratio of the AMPA receptor current to the NMDA receptor current (AMPA/NMDA) in VTA neurons, which allows for comparing the excitatory synaptic strength between different groups of animals (i.e., drug treated vs. control). *In vivo* exposure to drugs of abuse increases the AMPA/NMDA (202–204, 206, 207), which is mediated by insertion of calcium-permeable AMPA receptors and removal of NMDA receptors in VTA dopamine neurons (205, 208).

In addition to the excitatory synaptic alterations in VTA dopamine neurons, *in vivo* exposure to drugs also modulates inhibitory synaptic inputs to the VTA. For example, repeated injections of cocaine potentiate the NAc inhibitory input to VTA GABA neurons, which results in a disinhibition of dopamine neurons (179). This disinhibition also facilitates the ability to elicit excitatory long-term potentiation (LTP) in VTA dopamine neurons (209). VTA dopamine neurons are also capable of undergoing inhibitory LTP. Furthermore, this inhibitory LTP is blocked following an *in vivo* exposure to opiates (210, 211). A myriad of drug-induced synaptic alterations have been reported, though it is important to note that the full complement of electrophysiological changes and the duration of these alterations in VTA neurons depends upon the drug, the drug dose, and the manner the drug is administered (202–204, 206, 207, 212). Few studies to date have examined whether these drug-induced synaptic changes occur in an afferent-specific manner (179, 212). Indeed, *in vivo* exposure to different classes of abused drugs results in alterations in distinct excitatory inputs to VTA dopamine neurons (212). Although much has been learned regarding synaptic alterations in the VTA following non-contingent injections of abused drugs, additional studies are needed to ascertain the similarities and differences in the synaptic changes evoked by different classes of abused drugs (psychostimulants, opiates, alcohol, nicotine, etc.). Furthermore, electrophysiological studies are also needed to identify which VTA afferents and what VTA neuronal populations undergo synaptic alterations following contingent drug self-administration.

## Conclusion

The high incidence of relapse illustrates the need for identifying new therapeutic approaches for the treatment of SUDs. The treatment of opioid dependence is complicated by the severe withdrawal symptoms experienced by individuals when ceasing drug intake. The current treatment options for opioid SUDs typically focus on opioid maintenance with methadone or buprenorphine and detoxification with alpha-2 receptor agonists. However, these current treatment options often result in relapse (213). Currently there is no FDA-approved pharmacotherapy for the treatment of cocaine SUDs, though *N*-acetylcysteine is a promising and well-tolerated drug that reduces cocaine-seeking in rodents and craving in cocaine-dependent humans (214–217). Over the past decade, research on effective pharmacological treatments for alcohol SUDs has identified many potential targets, including opioid receptors (218), dopamine receptors (219), glutamate receptors (220), GABA receptors (221), and adrenergic receptors (222). Preclinical research highlighted the cannabinoid system as a promising target for multiple SUDs (223, 224). However, a cardiovascular clinical study examining the efficacy of rimanobant, a cannabinoid receptor antagonist, elicited severed negative neuropsychiatric effects (225) and has dampened enthusiasm for targeting the endocannabinoid system for treating SUDs. Unfortunately, no single pharmacotherapy currently exists for treating a broad spectrum of SUDs.

An alternative therapeutic direction for the treatment of SUDs involves the use of deep brain stimulation (DBS), which commonly has been utilized for the treatment of movement disorders. In preclinical studies, DBS targeting the NAc reduced cocaine behavioral sensitization (226), morphine CPP (227), reinstatement of heroin-seeking (228), and reinstatement of cocaine-seeking (229–231). Additionally, DBS targeting the LHb reduces cocaine self-administration and the reinstatement of cocaine-seeking (232). Consistent with the rodent DBS experiments, clinical studies indicate a complete remission or prolonged cessation of heroin use after DBS in the NAc in humans (233, 234). A considerable drawback of implementing DBS in humans is the invasive nature of implanting the probe. However, a couple of recent reports illustrate that non-invasive transcranial magnetic stimulation of the PFC is effective at reducing drug use and craving (235, 236). While there are promising new therapeutic approaches for treating SUDs, the ultimate goal for any intervention is to be effective and as specific as possible to limit side effects. Thus, additional basic science research is needed for identifying the specific neural circuits and adaptations responsible for the development of drug-dependent behaviors.

The implementation of optogenetic and chemogenetic approaches in behavioral experiments has validated and identified specific neural circuits that mediate a range of appetitive and aversive behaviors. Many of these studies manipulated brain regions implicated in SUDs (237), though relatively few have modulated neural circuits within the context of drug-dependent behaviors (98, 113, 133). While activity within the VTA is central to numerous drug-dependent behaviors, many questions remain. Future experiments are needed to (i) determine which VTA afferents and what neuronal populations in the VTA mediate a particular drug-dependent behavior and (ii) elucidate the associated afferent-specific synaptic changes on both dopamine and non-dopamine neurons within the VTA. Identifying the neural circuits and adaptations responsible for drug-dependent behaviors in rodents can highlight specific neural circuits for targeted pharmacological and DBS therapeutic interventions to treat humans suffering from a SUD.

## Author Contributions

MW and IO contributed to the writing of this review article.

## Conflict of Interest Statement

The authors declare that the research was conducted in the absence of any commercial or financial relationships that could be construed as a potential conflict of interest.
